# Multi-Sensor NDVI Time Series for Crop and Fallow Land Classification in Khabarovsk Krai, Russia

**DOI:** 10.3390/s25185746

**Published:** 2025-09-15

**Authors:** Lyubov Illarionova, Konstantin Dubrovin, Elizaveta Fomina, Alexey Stepanov, Aleksei Sorokin, Andrey Verkhoturov

**Affiliations:** 1 Computing Center of the Far Eastern Branch of the Russian Academy of Sciences, Khabarovsk 680000, Russia; illarionova_l@list.ru (L.I.); eliz37@mail.ru (E.F.); alsor@febras.net (A.S.); andrey@ccfebras.ru (A.V.); 2 Far-Eastern Agriculture Research Institute, Vostochnoe 680521, Russia; stepanfx@mail.ru

**Keywords:** crop mapping, time series, function-fitting, optical remote sensing, multi-sensor

## Abstract

Automatic cropland monitoring is becoming increasingly important in the advancement of sustainable agriculture. However, multiannual satellite-based crop mapping across different regions remains challenging due to variations in crop phenology and meteorological conditions. The use of multispectral data from a single satellite can also present difficulties in constructing vegetation index time series, particularly in regions affected by persistent cloud cover. In this study, NDVI time series obtained from Sentinel-2 and Landsat-8/9 imagery were fitted using a Fourier series, and daily NDVI composites from the Meteor-M satellite were obtained for Khabarovsk Krai in the Russian Far East from 2022 to 2024. These data were used to perform random forest (RF) classification for each year for five land cover classes: soybean, grain crops, perennial grasses, buckwheat, and fallow land. The average annual classification accuracies were 87% for Landsat 8/9, 89% for Meteor-M, and 93% for Sentinel-2. Combining data from all three satellites improved classification performance, increasing cross-validation overall accuracy from 92% to 96% in 2022, from 96% to 97% in 2023. These results demonstrate the potential of using both individual satellite data for sufficiently accurate mapping and combined datasets, which provide consistently high accuracy and are a reliable alternative when Sentinel data are limited due to cloud cover.

## 1. Introduction

Innovative solutions are at the forefront of technological advancement, driving unprecedented progress in various sectors, particularly in improving food security [1]. These technologies have the potential to revolutionize global food production through advancements in precision farming, smart farming practices, automation, and monitoring technologies [2,3,4]. Remote sensing is used for various agricultural tasks, such as crop recognition, land cover classification, crop health assessment, and yield prediction, due to its global coverage and high data availability [5,6,7].

Landsat-8/9 and Sentinel-2 are the most widely used satellite systems for land surface monitoring. Landsat-8/9, which offers a spatial resolution of 30 m, is extensively used for agricultural monitoring [8,9]. The imaging frequency of a single Landsat satellite is 16 days, which is reduced to 8 days when two satellites are used concurrently [10]. The Sentinel-2 satellite constellation, developed by the European Space Agency (ESA) and comprising Sentinel-2A, 2B, and 2C, provides accurate, real-time land surface data for vegetation mapping and monitoring, as well as crop health assessment. These satellites collect imagery across 13 distinct spectral bands (ranging from 443 nm to 2190 nm), with spatial resolutions of 10 m, 20 m, and 60 m, depending on the band, and a temporal frequency of 5–10 days [11].

The Russian orbital constellation for remote sensing supports tasks related to monitoring the Earth’s surface. Meteor-M satellites are among the medium spatial resolution systems. The polar-orbiting satellites in this series are designed to provide operational data to the Russian Federal Service for Hydrometeorology and Environmental Monitoring, research institutes, and other organizations [12]. These satellites are used to observe objects and phenomena in the atmosphere, hydrosphere, and cryosphere for global climate monitoring, emergency response, and environmental monitoring [13]. Currently, four Meteor-M satellites–Meteor-M No. 2, No. 2.2, No. 2.3, and No. 2.4—are operational in orbit. The launch of three additional satellites is planned. The Meteor-M No. 2 satellite, equipped with the Multispectral Satellite Imaging System (KMSS-M) instrument, was launched on 8 July 2014, and placed into a sun-synchronous orbit at an altitude of approximately 830 km [14]. The KMSS-M system includes two optical cameras, MSD-201 (Multispectral Scanning Device) and MSD-202, which are designed to perform measurements in three spectral bands—green, red, and near-infrared (NIR)—with a spatial resolution of 60 m. Meteor-M No. 2.2, launched on 5 July 2019, carries the KMSS-2 instrument [13]. Meteor-M No. 2.3 was launched on 27 June 2023, followed by Meteor-M No. 2.4 on 29 February 2024. KMSS-M data are widely used for remote assessment of land cover characteristics and for operational monitoring in Russia and surrounding regions [15,16].

Spectral radiance values from individual satellite image bands are used to analyze vegetation types and conditions. Denser and healthier vegetation is characterized by a greater contrast in reflection in the red and NIR bands. Vegetation indices, such as normalized difference vegetation index (NDVI), soil-adjusted vegetation index (SAVI), and enhanced vegetation index (EVI), are based on this principle and are extensively applied in cropland mapping [17]. When monitoring cropland, it is important to assess the temporal dynamics of vegetation indices throughout the growing season and construct seasonal time series [8,18,19]. Constructing a vegetation index time series involves compiling a series of satellite images captured during the growing season. However, atmospheric phenomena, such as cloud cover and cirrus, can significantly degrade optical data quality. Therefore, the careful selection of suitable images is critical for time series construction and accurate cropland mapping. Common approaches involve building time series using only cloud-free images, such as those with scene cloud cover less than 1% [20] or less than 5% [21], or using images with low scene cloud cover (less than 20%) [22,23,24]. However, extended periods of cloudiness during the growing season are typical in several regions worldwide, including the Russian Far East. This may lead to insufficient availability of satellite images for constructing a continuous time series. In such cases, missing values can be estimated using interpolation techniques and filtering methods [25,26,27]. When time series data are significantly sparse, function-fitting methods are often used instead of interpolation. For NDVI time series fitting, functions that resemble the typical NDVI seasonal curve are most commonly used. These include polynomial functions [5], the Gaussian function [28], the double logistic function [29], and the Fourier series [30].

Using data from multiple satellites both increases the number of observations during the growing season, particularly in regions with significant cloudiness, and aids in analyzing vegetation index values derived from different sensors, which may have varying spectral bandwidths for the same bands. This approach also enables the comparison of the effectiveness of different satellite systems for vegetation monitoring tasks. In the case of increasing observational frequency, a single time series is constructed for each sample by integrating imagery from multiple satellites. For instance, crop maps of Germany with 24 classes from 2017 to 2019 were generated using time series created from both Sentinel-2 and Landsat-8 data [31]. The optical data included both spectral bands (e.g., RGB, NIR, SWIR) and indices (e.g., NDVI, NDWI, SAVI). The Landsat-8 and Sentinel-2 channels were sharpened to a 10 m resolution and aligned to the Sentinel-2 grid. A convolution-based interpolation filter was applied to fill gaps in the time series and generate 5-day composite images. The random forest (RF) algorithm was used as the classifier. A total of 863 pixels were selected for training and 870 pixels for testing, ensuring proportional class representation and spatial distribution. Due to the large number of features used, the size of the training and test samples was too small, resulting in low recognition accuracy. The classification accuracy ranged from 67 to 70%. Berra et al. propose a methodology for creating harmonized Landsat-7/8 and Sentinel-2 time series using Google Earth Engine (GEE), outlining the key steps for constructing continuous time series at a 30 m resolution [32]. A coefficient of determination of 0.98 was observed between the harmonized time series and ground-based NDVI measurements, confirming the viability of such data for monitoring applications. Tian et al. used high-resolution imagery from Gaofen-2 and Jilin-1 satellites to obtain field boundaries [33]. Sentinel-2, ZY-1 images, and DEM elevation data were used for crop classification in mountainous regions of China, such as Xishui County in Guizhou Province, where field sizes are typically small. A total of 798 points were selected for training and 1200 for testing. Pixel-level classification accuracy using the RF algorithms was not high—only 75% (70% for maize), field-level classification (aggregated from the pixel classification results) achieved 85% accuracy (84% for maize).

Products that combine Landsat-8 and Sentinel-2 data, such as the Harmonized Landsat Sentinel-2 (HLS) dataset [34], are widely used for crop monitoring. The HLS provides a 30 m spatial resolution with a revisit interval of 2–3 days. For instance, an algorithm was developed for the early detection of soybean and corn crops in the United States Corn Belt, examining several weeks of the growing season [35]. To achieve this, two vegetation indices were created, and field-level time series were constructed using data from HLS, The Suomi National Polar-orbiting Partnership (Suomi-NPP), and Operational Environmental Satellite-R series (GOES-R) satellites. Suomi and GOES data (600 and 1000 m resolution, respectively) were combined using the Spatiotemporal Shape-Matching Model (SSMM) algorithm. Three-day composites for Suomi and GOES were first constructed separately for the two years preceding the assessment date and then combined with Landsat-Sentinel data. Similar pixel values from 2018 to 2019 within a 5 km radius were used to estimate missing values for 2020. Phenometrics were extracted from the time series using the Gaussian Mixture Model (GMM) model. Following the separation of other crop types, binary classification was performed for the period between 30 May and 28 August. By the end of July, crop identification accuracy exceeded 90%.

In several previous studies, classification datasets were created individually for each satellite. For example, Landsat-8 and Sentinel-2 images were used to construct crop maps in Uzbekistan for the year 2018 [36]. Monthly composites of four vegetation indices were used: NDVI, EVI, Normalized difference water index (NDWI), and NDWI2. RF and support vector machine (SVM) algorithms were applied as machine learning methods. Classification was performed separately for each index and satellite. The NDVI-based classification accuracy was 85% for Landsat-8 data when using RF and 87% for Sentinel-2 data when using SVM. The highest accuracy of 90% was achieved using Landsat-8 EVI data. Similarly, Wang et al. performed crop identification at 12 sites in Henan Province, China, using Sentinel-2 and Gaofen panchromatic and multispectral sensor (PMS) images from 2019 to 2021, using a neural network based on the UNet++ architecture [37]. Three images from each satellite, acquired in April of each year, were used. Data from 2021 were excluded from training. Both individual bands and vegetation indices were used. The classification accuracy using Sentinel-2 was 97%, with an F1 score of 71%, while that using Gaofen imagery ranged from 75 to 97%, with F1 scores ranging from 55% to 73%.

However, the use of data from only one of the satellite systems in the case of regions with a large number of cloudy days during the growing season (such as Khabarovsk Krai) may lead to a lack of images to analyze the vegetation process, and therefore to a significant inter-annual variation in the accuracy of crop and fallow land mapping. Despite the extensive research conducted across various countries, developing a method that integrates data from multiple satellites to construct stable classification models capable of producing continuous time series and maintaining high classification accuracy over the long term remains an urgent challenge. Therefore, the objective of this study is to develop an effective method for identifying crops and fallow land at the district level using imagery from three satellite systems (Sentinel-2, Landsat-8/9, and Meteor-M) and an algorithm for building continuous NDVI time series composites. To achieve this objective, the following tasks were performed: construction and analysis of NDVI reference time series for the region’s main crops and fallow land; evaluation of crop recognition accuracy using data from different satellite systems; development of a composite classification algorithm using data from three satellite systems; and assessment of the overall classification accuracy. The proposed method was tested for generating crop maps in the Khabarovsk District of Khabarovsk Krai in the Russian Far East.

## 2. Materials and Methods

### 2.1. Study Area and Ground Truth Data

**Figure 1:**
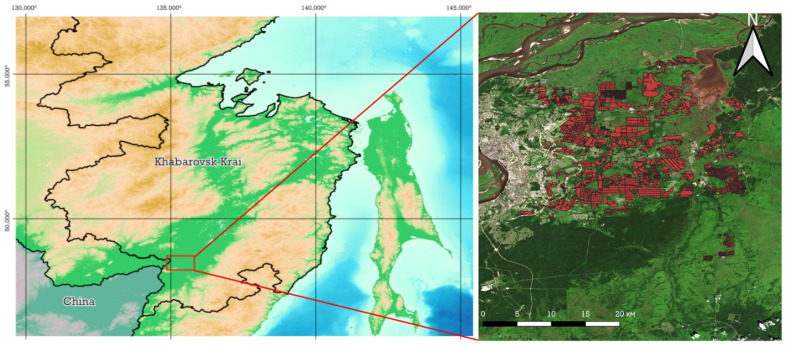
Study area.

The study was conducted in the southeastern part of the Khabarovsk District in Khabarovsk Krai, on the right bank of the Amur River between 48.31° and 48.64° N latitude and 134.81° and 135.57° E longitude in the suburbs of Khabarovsk (Figure 1), from 2022 to 2024.

In this study, crop rotation data were used for 421 fields in 2022, 462 fields in 2023, and 996 fields in 2024. The total area covered by these fields was approximately 36,052.04 ha, and all fields were categorized into one of five classes: soybean (20,384.08 ha), fallow land (7001.16 ha), perennial grasses (2623.88 ha), grain crops, such as wheat, oat, and barley (4620.84 ha), and buckwheat (1422.08 ha). Statistical information for municipal districts of Khabarovsk Krai is not complete and field borders are not always reliable. That is why field boundaries and crop rotation data were validated through ground observations and visual analysis of Sentinel-2 images Table 1 presents the distribution of areas across these crops and fallow land categories. It is important to note that only spring crops are cultivated in the Khabarovsk Krai. Sowing typically begins in early May, and harvesting continues until the end of October.

### 2.2. Study Flowchart

**Figure 2:**
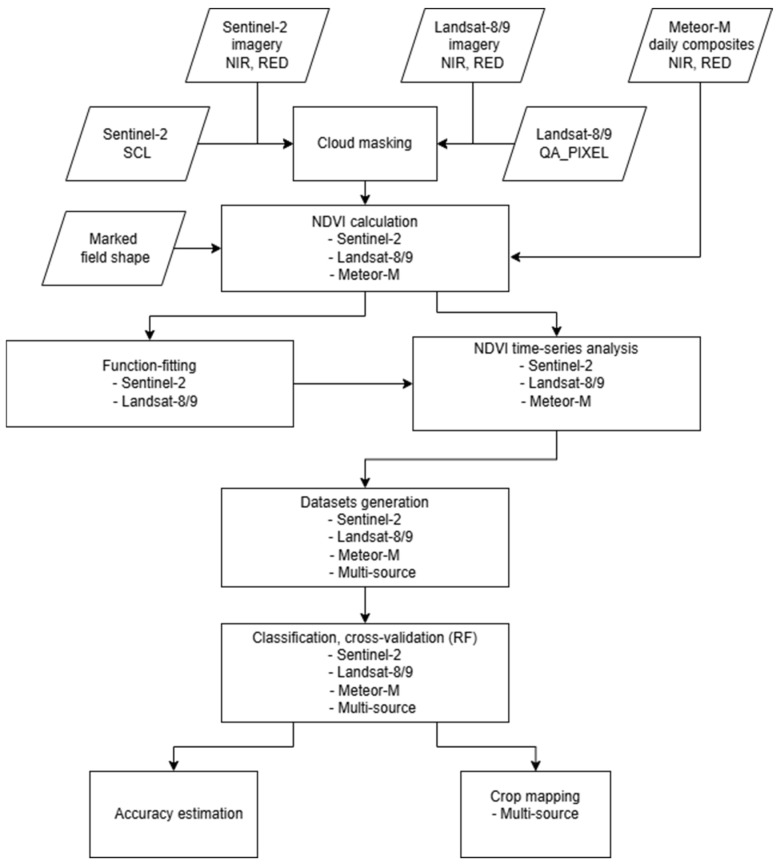
Study flowchart.

NDVI time series were constructed through a series of steps, including cloud masking, NDVI calculation, and function fitting. This was followed by machine learning-based classification, cross-validation, and the generation of cropland maps. Figure 2 presents an overview of the research design.

### 2.3. Sentinel Data

For the study area, the following datasets were considered:

- Daily composite images from the Meteor-M No. 2 satellite (hereinafter referred to as “Meteor”) in the NIR and red bands, covering days 122 to 269 of the calendar year (DOY), corresponding to early May through late September. These images have a 60 m resolution and were obtained from the Center for Collective Use “Space Research Institute-Monitoring” (CCU “IKI-Monitoring”) [38,39].
- A total of 181 images with Level 2A processing from the Sentinel-2A/B satellites (hereinafter referred to as “Sentinel”), with a spatial resolution of 20 m.
- A total of 47 Level 2A processed images from the Landsat-8/9 satellites (hereinafter referred to as “Landsat”), with a spatial resolution of 30 m.

To create cloud masks for each Sentinel polygon, the scene classification (SCL) product was used. Pixels with values of 0 (no data), 1 (saturated or defective), 3 (cloud shadows), 8 (medium probability of clouds), 9 (high probability of clouds), 10 (haze), and 11 (snow) were marked as unsuitable for analysis and masked. For Landsat imagery, the Pixel Quality Assessment (QA_PIXEL) band was used for cloud masking. Only pixels with a QA_PIXEL value equal to 21,824, indicating the absence of haze, clouds, cloud shadows, snow, and water, were considered valid; all others were masked.

### 2.4. NDVI Time Series Construction

The authors’ software “Program complex of automated processing of Meteor satellite composite images for obtaining seasonal NDVI time series for cropland of Khabarovsk Krai” [40] was used to select pixels within field boundaries from satellite images, generating CSV files for each field. These files stored geographic coordinates and the calculated NDVI values for each observation date. The NDVI was calculated using the standard formula:

(1) $$NDVI\;=\frac{NIR-RED}{NIR+RED},$$

where *NIR* represents reflectance in the *NIR* region of the spectrum and *RED* represents reflectance in the red region of the spectrum.

Image processing was performed using the Rasterio [41] and GDAL [42] libraries in Python 3.11.9. For each pixel in the study area, weekly NDVI composite time series were constructed for the vegetation seasons of 2022, 2023, and 2024. A major challenge in creating these composites was cloud cover. Table 2 summarizes the quality of the Landsat and Sentinel imagery over the study period, expressed as the proportion of masked pixels. More than half of the Sentinel and Landsat images were affected by cloud cover, including cloud shadows and cirrus. The number of low-cloud Sentinel images (defined as images having less than 20% masked pixels) was 13 in 2022 (21% of the total), 15 in 2023 (25%), and 14 in 2024 (24%). However, the number of truly cloud-free images (defined as having less than 5% masked pixels) that were suitable for use without data restoration was even smaller and unevenly distributed throughout the growing season. For example, in 2022, only eight Sentinel-2 images satisfied this condition, half of which were acquired in September, with no cloud-free images available in June. In 2023, 11 cloud-free images were obtained, including four in July and one each in May and August. In 2024, another 11 cloud-free images were captured, with four in September and one each in June and August. This uneven temporal distribution of cloud-free and low-cloud images limited the feasibility of constructing weekly NDVI composites from Sentinel data. In many weeks, no usable images were available, while some weeks had two images. This data deficiency issue was even more pronounced for Landsat data due to the lower frequency of image acquisition (14–18 per season). Across all three years, only five Landsat images were cloud-free, and an additional six images were classified as low-cloud, with 5–20% of pixels masked.

Thus, to generate continuous time series despite gaps in the data, NDVI values were estimated using function fitting. Specifically, to obtain daily values within the specified time period, the time series was fitted using the first two terms of a Fourier series decomposition [43], given by:

(2) $$f=a_{0}+a_{1}\times cos\left(xw\right)+b_{1}\times sin\left(xw\right)+a_{2}\times cos(2xw)+b_{2}\times sin(2xw),$$

where $w,\;a_{0},\;a_{1},\;a_{2},\;b_{1},$ and $b_{2}$ are the fitting parameters.

Through this approach, continuous weekly NDVI composites were derived for each pixel for DOY 121 to 296 for both Landsat and Sentinel data. In addition, NDVI values for Meteor data were calculated using Equation (1), based on daily composite images of the NIR and RED bands. The weekly composites were generated using the sliding window method, ensuring a uniform spatial and temporal resolution across all datasets to facilitate comparison. The obtained data were then used for statistical analysis and classification.

Datasets from Landsat, Sentinel, and Meteor satellites for the period 2022–2024 were created using synchronized NDVI data. Automated co-registration, defined as the selection of a single reference point and coordinate system, and an image resampling were performed using the Rasterio and GDAL libraries in Python 3.11.9. As a result of this preprocessing, the pixel coordinates across all satellite images were aligned. Subsequent steps included preprocessing and time series function fitting using Fourier series fitting, as previously described. For each co-registered pixel, a set of three NDVI values per week, corresponding to Landsat, Sentinel, and Meteor data, was generated. These values were then matched to the Meteor coordinate grid, resulting in a harmonized dataset with a spatial resolution of 60 m.

#### Multi-Source Data Combination Method

When processing remote sensing data (Sentinel and Landsat), a key step is to convert images to a single coordinate system and spatial resolution. The presented methodology uses the rasterio.warp package, which performs joint conversion of data to the WGS84 (EPSG:4326) coordinate system with a decrease in resolution to 60 m. The process is implemented through three interrelated stages.

The creation of the target spatial grid is initiated by defining the parameters of the output raster. Based on the target EPSG:4326 coordinate system and the specified resolution of 60 m, a regular geographic grid is generated. The pixel size in degrees is automatically calculated taking into account the sphericity of the Earth, amounting to approximately 0.00054°, which corresponds to the metric equivalent of 60 m at the equator (based on the ratio 1° ≈ 111 km). This conversion ensures the geometric correctness of subsequent transformations.

Data resampling is performed using bilinear interpolation, which is optimal for continuous geophysical parameters. The algorithm calculates the value of each pixel in the output raster as the weighted average of the four nearest pixels in the original image. The weighting coefficients are determined by the Euclidean distance to the target position, which ensures smooth transitions of values and minimizes discreteness. For Sentinel-2 data with an original resolution of 20 m, conversion to 60 m pixels takes into account the spatial distribution of the nine original elements through the interpolation of four key points. Similarly, for Landsat images with a native resolution of 30 m, each new pixel integrates the information of four adjacent elements of the original matrix.

Reprojection into the target CRS is implemented through inverse coordinate transformation. For each pixel of the generated EPSG:4326 grid, the corresponding position in the original coordinate system (e.g., UTM of a specific zone) is calculated. The transformation is performed using affine transformations that take into account the ellipsoidal model of the Earth. The value of the resulting pixel is determined by bilinear interpolation from the neighborhood of the calculated point in the original raster, which guarantees geodetic accuracy and preservation of spatial relationships.

Performing resampling and reprojection together minimizes resampling errors and ensures consistency in the spatial characteristics of heterogeneous satellite data.

### 2.5. Construction and Analysis of NDVI Reference Time Series

In the subsequent stage of the analysis, the mean NDVI values across all pixels within each field were calculated. Two key indicators derived from the fitted time series were used as primary features for classification: ${NDVI}_{max}$, which was the averaged field NDVI maximum value, and ${DOY}_{max}$, representing the day of the year corresponding to ${NDVI}_{max}$. To generate mean NDVI time series for each crop class, field-level NDVI time series were averaged across all fields belonging to a given class. Subsequently, both the maximum NDVI value and the day it occurred were extracted, and the variability of these features was analyzed. To evaluate the differences in NDVI characteristics over the study period, a one-way analysis of variance (ANOVA) was performed. Pairwise comparisons were performed using Tukey honest significant difference (HSD) test.

### 2.6. Crop Mapping and Accuracy Estimation

The crop classification process was performed using the RF algorithm, as implemented in the scikit-learn library [44] in Python 3.11.9. The model was configured with 50 decision trees. Data were partitioned into a training and test set using the GroupShuffleSplit method from the scikit-learn library. This method enables data splitting while accounting for group membership, where each field represents a distinct group, and helps preserve the original crop area ratio, which is a critical consideration when working with imbalanced datasets. The test set comprised 50% of the total dataset.

Prior to classification, the training data were filtered to remove outliers based on the interquartile range (IQR) for each individual crop class. The outlier thresholds were determined:

(3) $$\left[Q_{1}-1.5\times IQR,\;Q_{3}+1.5\times IQR\right],$$

where $Q_{1}$ represents the first quartile of the dataset, $Q_{3}$ represents the third quartile, and $IQR\;=\;Q_{3}-Q_{1}$.

Cropland classification in the Khabarovsk District for the years 2022–2024 was conducted using NDVI time-series datasets from Sentinel, Landsat, Meteor, and a multisource dataset. Crop maps were constructed using the multi-sensor approach.

The use of ground observation data allows the accuracy to be assessed. For all polygons it was estimated how many pixels fell into the class specified for the polygon (based on field observations). Model accuracy was evaluated using confusion matrices, where rows represent actual classes and columns represent predicted classes, overall accuracy (OA), and the F1 score for each class. These metrics were calculated using the following equations:

(4) $$OA\;=\frac{TP+TN}{TP+TN+FP+FN},$$

(5) $${F1}_{i}=\frac{{TP}_{i}}{{TP}_{i}+\frac{{FP}_{i}+{FN}_{i}}{2}},$$

where *TP*, *TN*, *FP*, and *FN* refer to the number of true positives, true negatives, false positives, and false negatives, respectively, and *i* represents the index of the crop class.

To further assess model quality, cross-validation was performed using the StratifiedGroupKFold method from the scikit-learn library. This technique involved the division of the dataset into three distinct folds using a stratified K-fold iterator while ensuring that groups do not overlap. During partitioning, all data points from a given field were assigned to the same fold, thereby implementing a field-based split. Each field was included in the training set only once, and the proportion of fields from each class was maintained to reflect the original class distribution. OA and the mean F1 score (${F1}_{mean}$) were used as cross-validation accuracy metrics. ${F1}_{mean}$ was calculated as the average F1 score across all classes.

## 3. Results

### 3.1. Reference NDVI Curves for the All Classes

This section presents the averaged NDVI time series for the main crop types cultivated in Khabarovsk Krai—soybean, grain crops, buckwheat, and perennial grasses—as well as fallow land, for the years 2022–2024, based on imagery from Sentinel, Landsat (following data recovery using function fitting), and Meteor.

**Figure 3:**
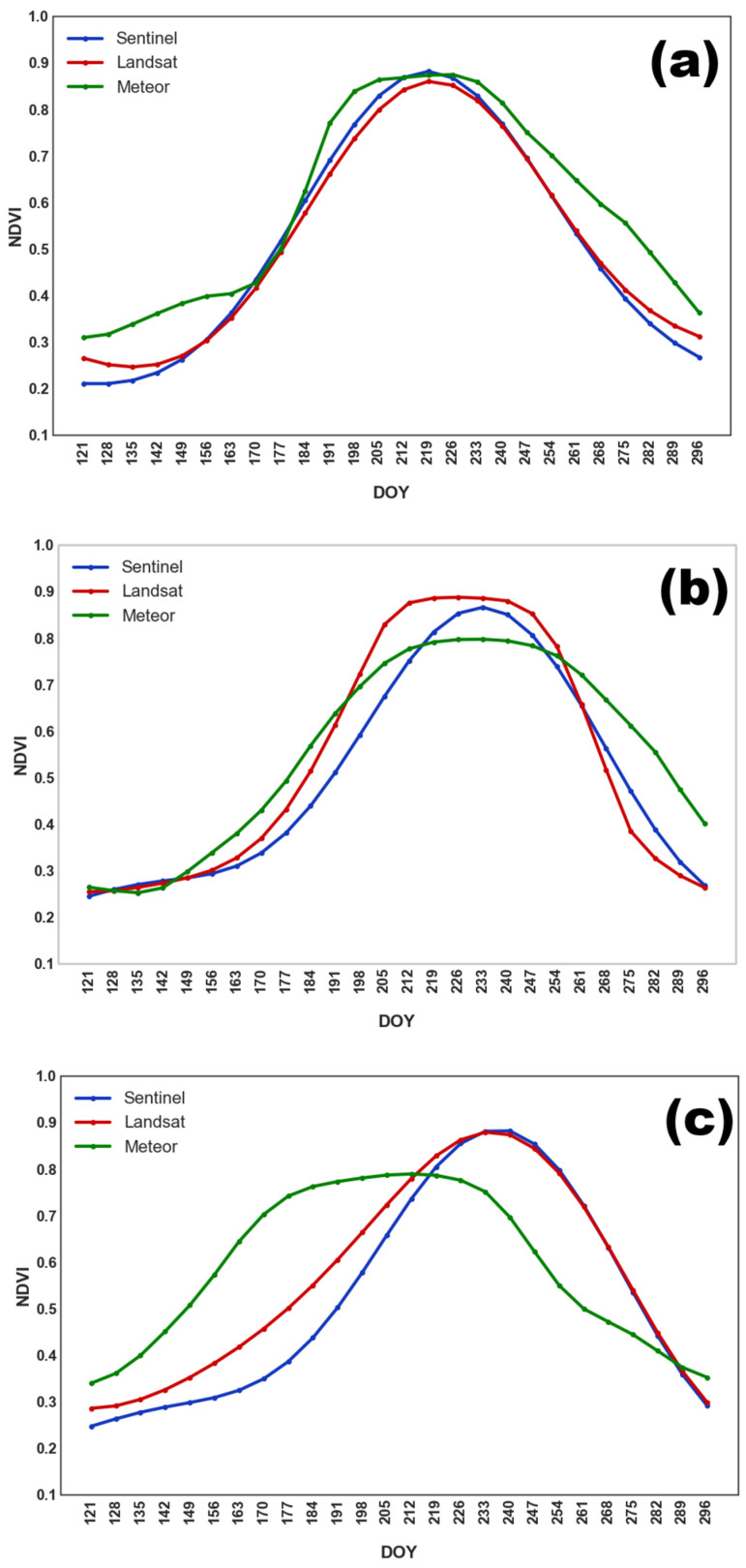
Averaged NDVI seasonal course for soybean obtained from different satellite systems in (**a**) 2022; (**b**) 2023; and (**c**) 2024.

The seasonal NDVI profiles for soybean (Figure 3) reflect the main stages of crop development. Soybeans were sown later than other crops (except buckwheat), from late May to mid-June. The NDVI maximum was observed between late July and late August (DOY 205–240). A gradual decline in NDVI followed, associated with green mass loss, until late September.

**Figure 4:**
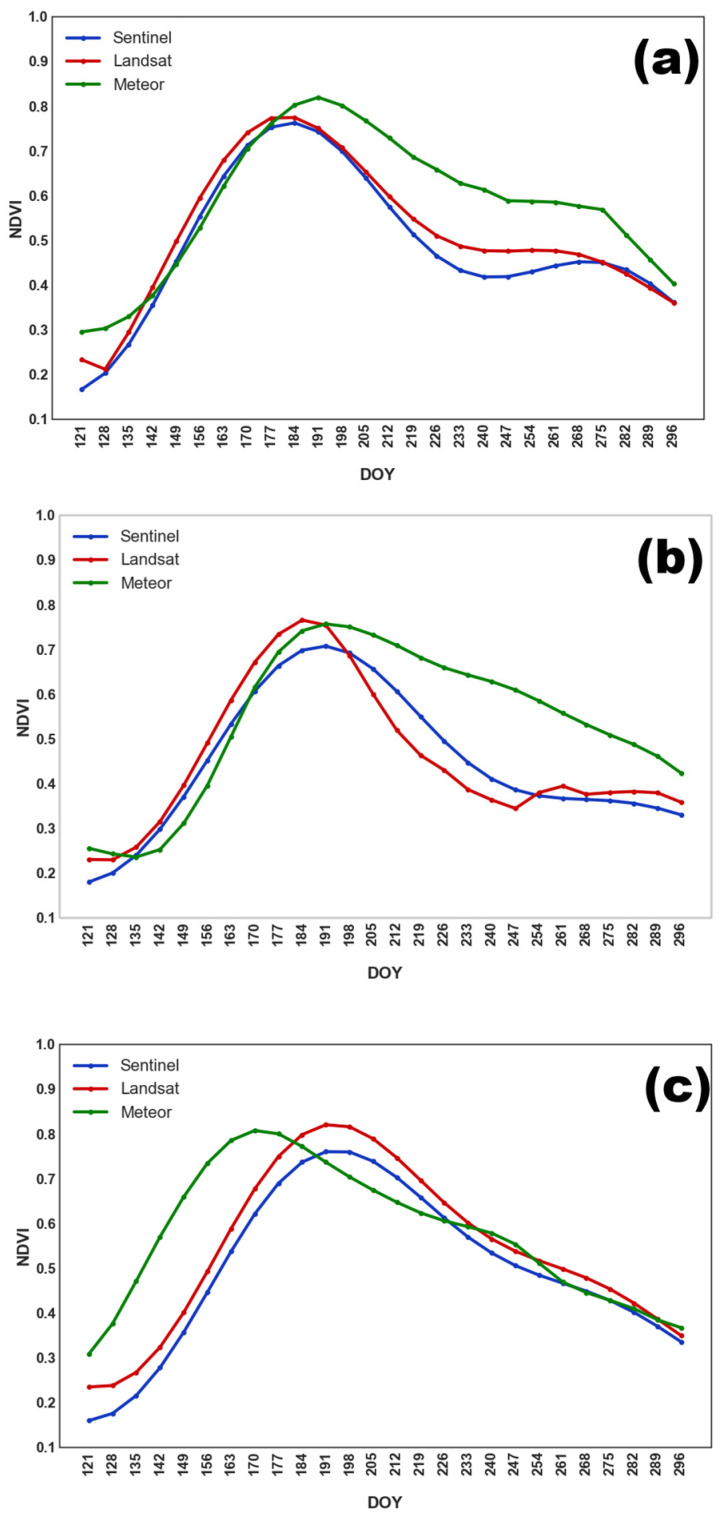
Averaged NDVI seasonal course for grain crops obtained from different satellite systems in (**a**) 2022; (**b**) 2023; and (**c**) 2024.

Grain crops, such as oat, wheat, and barley, exhibited similar NDVI dynamics, justifying their aggregation into a single class. As shown in Figure 4, NDVI peaked earlier than in soybean fields, with maximum values occurring between DOY 190–205 (mid to late July). This was followed by a decline in NDVI due to harvesting.

**Figure 5:**
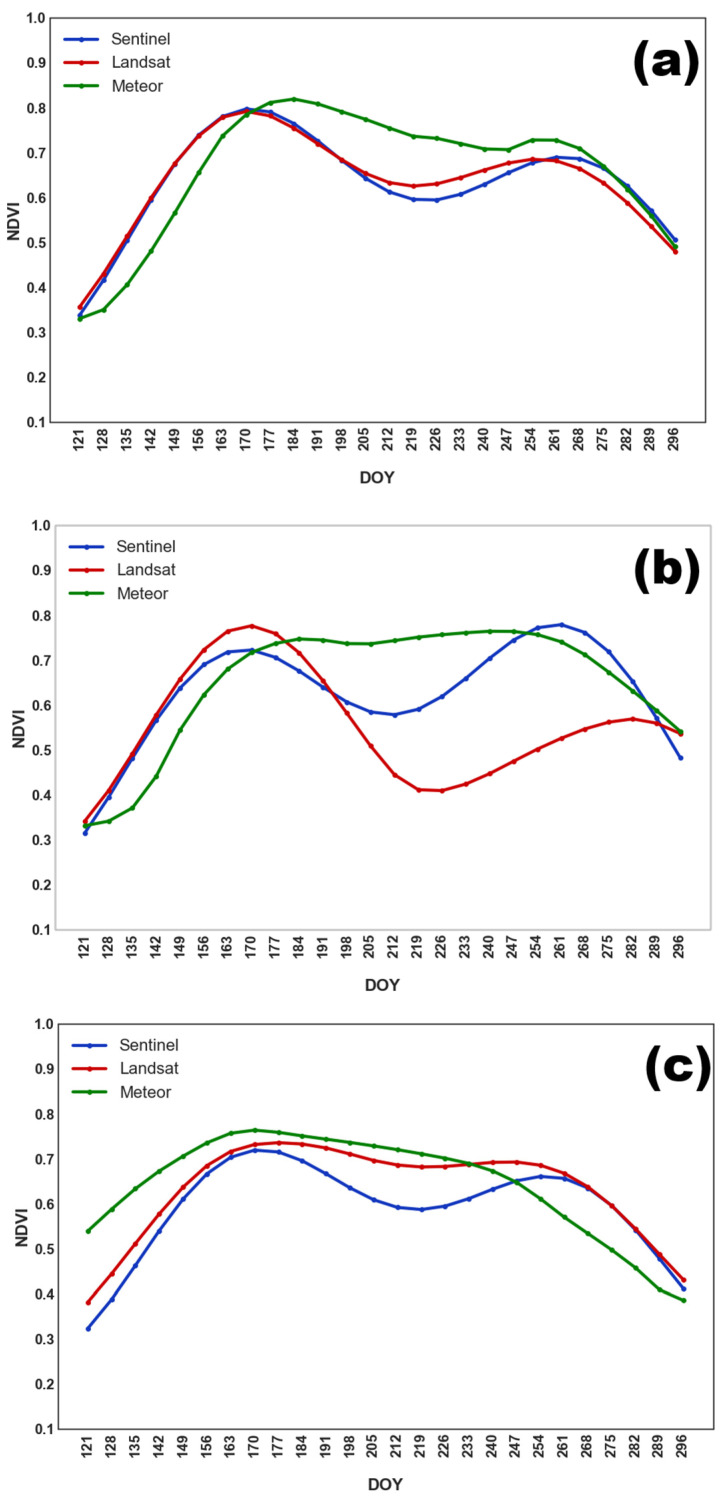
Averaged NDVI seasonal course for perennial grasses obtained from different satellite systems in (**a**) 2022; (**b**) 2023; and (**c**) 2024.

Similarly to grain crops, perennial grasses were a heterogeneous class consisting of timothy grass, clover, and other grasses. As a result, harvesting was carried out on different dates. However, NDVI values typically increased until late June or early July, followed by a second peak at the end of September. In most cases, a second mowing was not performed. The averaged NDVI seasonal profile for perennial grasses is shown in Figure 5.

**Figure 6:**
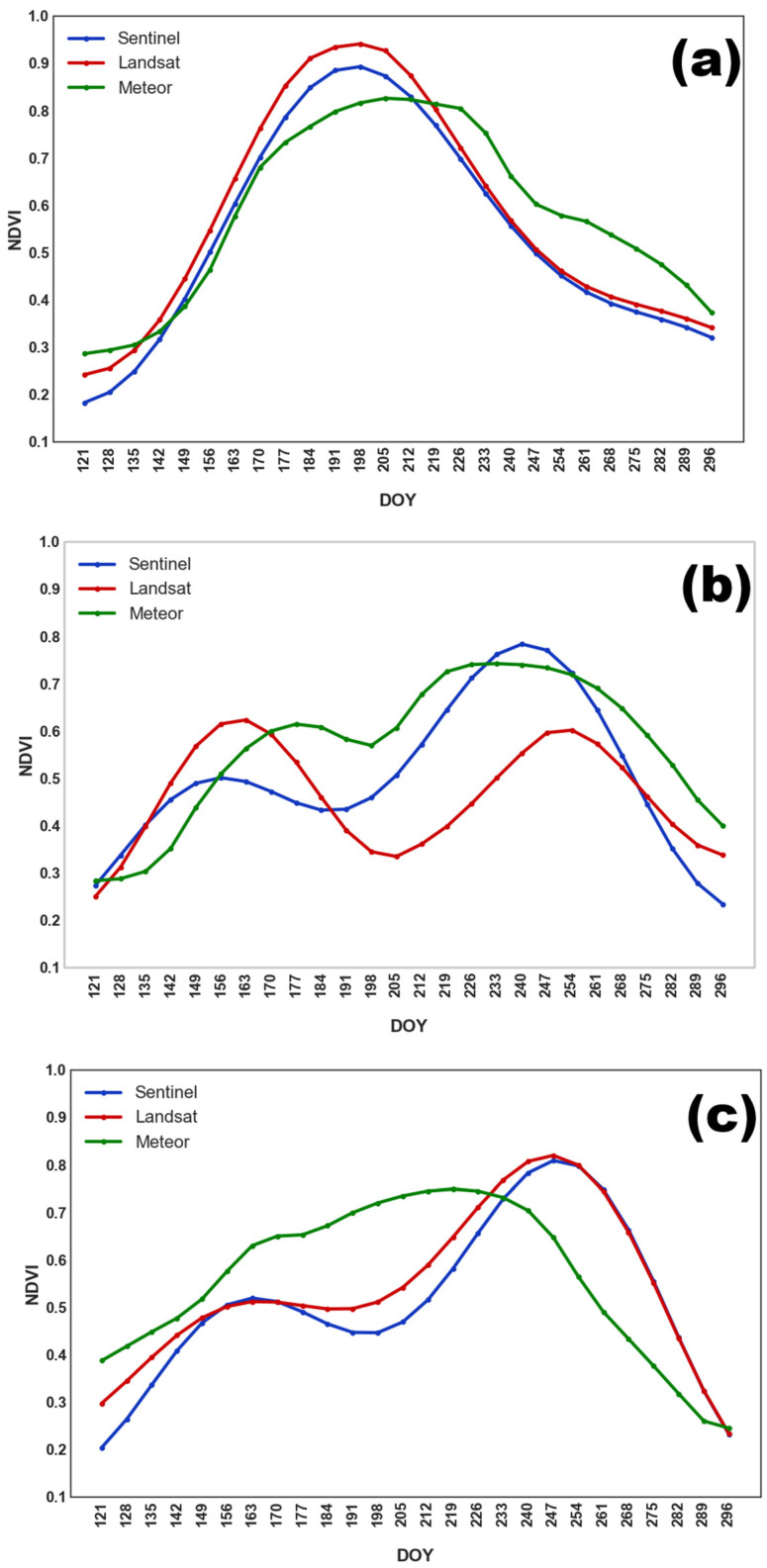
Averaged NDVI seasonal course for buckwheat obtained from different satellite systems in (**a**) 2022; (**b**) 2023; and (**c**) 2024.

In 2022 (Figure 6a), early sowing of buckwheat resulted in a single NDVI peak in late July, followed by a gradual decline. In contrast, the 2023 and 2024 seasons (Figure 6b,c) displayed two distinct peaks: an initial rise before plowing the field, and a second maximum in August or September, following sowing in July.

**Figure 7:**
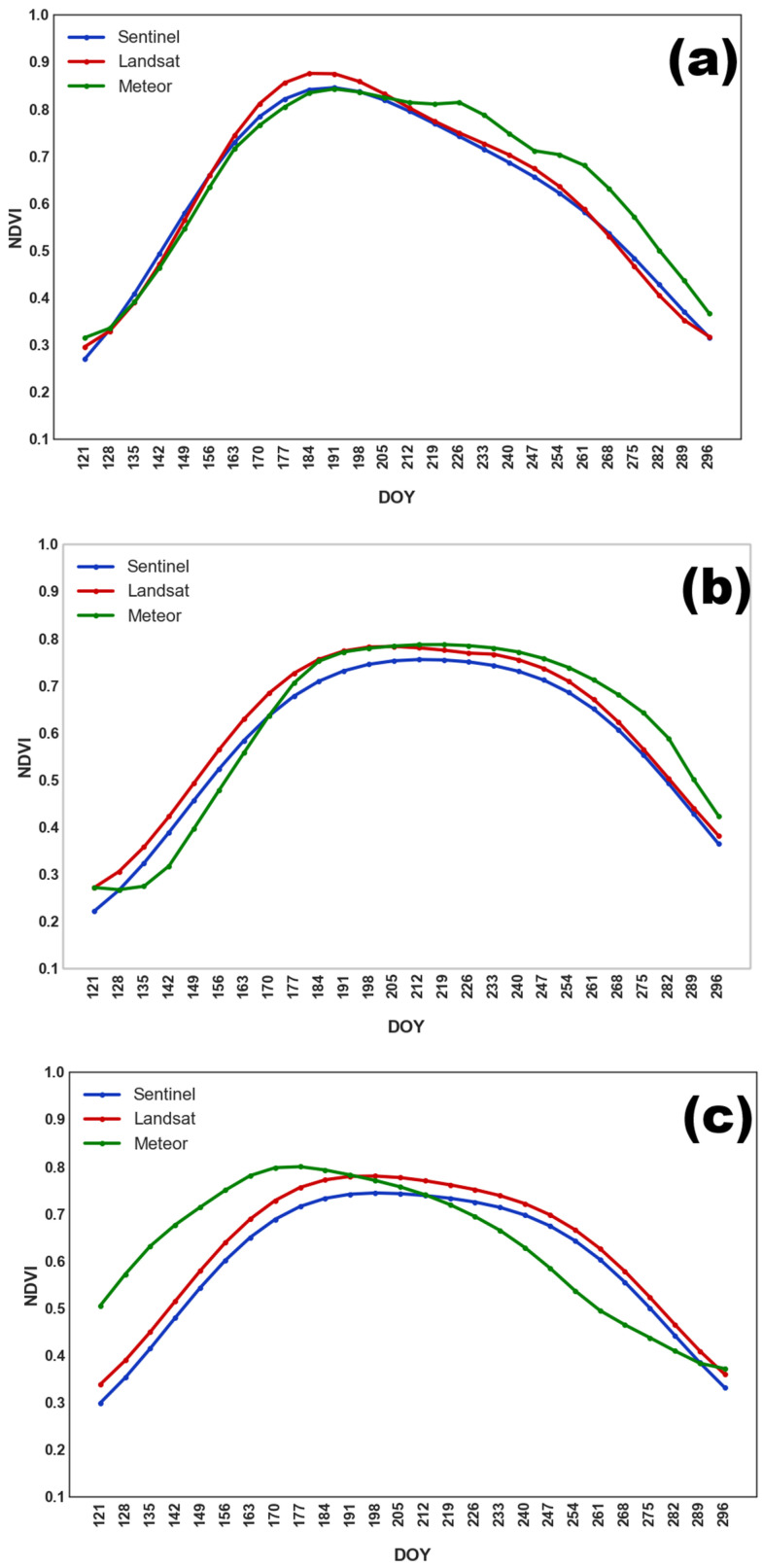
Averaged NDVI seasonal course for fallow land obtained from different satellite systems in (**a**) 2022; (**b**) 2023; and (**c**) 2024.

The NDVI profile for fallow land (Figure 7) exhibited a characteristic plateau shape, with a gradual increase in biomass leading to a maximum in July, followed by a steady decline in NDVI values until the end of the season.

Table 3 presents the mean NDVI maximum values ($\bar{{NDVI}_{max}}$), the mean dates of maximum NDVI ($\bar{{DOY}_{max}}$), and the associated variability of these indicators for soybean fields across different years. For Sentinel data, $\bar{{NDVI}_{max}}$ ranged from 0.87 to 0.89, with a variation of 5–7%. Landsat data yielded the highest average NDVI maximum in 2023 ($\bar{{NDVI}_{max}}\;$ = 0.91), with $\bar{{DOY}_{max}}$ occurring in August. In contrast, the Meteor-derived composites consistently showed lower $\bar{{NDVI}_{max}}$ values (0.8–0.81) with some variation between 2022 and 2023, and a tendency for the maximum to occur earlier in the season. For example, in 2024, $\bar{{NDVI}_{max}}\;$ occurred on DOY 203, corresponding to the third decade of July.

The results of ANOVA analysis and Tukey’s pairwise test revealed statistically significant variations in the timing of NDVI maxima ($\bar{{DOY}_{max}}$) for soybeans across the three years and all satellites (*p* < 0.001). However, no significant differences in NDVI peak values ($\bar{{NDVI}_{max}}$) were found between 2022 and 2023 or 2022 and 2024 for Sentinel, and between 2022 and 2024 for Landsat (*p* > 0.05).

Table 4 presents the NDVI time series indicators for grain crop fields, based on data from multiple satellite sources for 2022–2024. It is evident that the ${NDVI}_{max}$ values for grain crops were consistently lower than those observed for soybean crops. Additionally, greater variability in ${NDVI}_{max}$ was observed for grain crops, particularly in 2023 and 2024, when variability reached 9–10% for both Sentinel and Landsat data.

Among the satellite sources, Sentinel data, which has the highest spatial resolution, recorded the lowest average NDVI maxima (0.75–0.77). In contrast, Landsat-derived values were higher (0.8–0.83). For both satellite systems, the $\bar{{NDVI}_{max}}$ value occurred at analogous times: during the first decade of July in 2022 and 2023, and shifted to the third decade of July in 2024 (*p* < 0.05). The years 2022 and 2024 also exhibited substantial variability in maximum NDVI values, reaching up to 15%.

The NDVI series derived from Meteor-M imagery was characterized by lower variability in both ${NDVI}_{max}$ (3–6%) and ${DOY}_{max}$ (3–4%). Notably, in 2024, the maximum NDVI for Meteor was recorded on DOY 173 (i.e., the third decade of June), which is approximately one month earlier than for Sentinel and Landsat, and three weeks earlier than in 2022 and 2023 (*p* < 0.0001).

The results of the ANOVA and post hoc Tukey test revealed no statistically significant differences in ${NDVI}_{max}$ between years for both Landsat and Sentinel (*p* > 0.05). However, for Meteor, $\bar{{NDVI}_{max}}$ was 0.83 in 2022, which was significantly higher than the corresponding values observed in 2022 and 2023 (*p* < 0.0001).

For perennial grasses, $\bar{{NDVI}_{max}}$ ranged from 0.77 to 0.86, with the highest value recorded for Sentinel in 2023, and the lowest for Meteor in 2024. The occurrence of ${NDVI}_{max}$ differed depending on mowing schedules; fields with summer mowing reached maximum NDVI in the second half of June, while fields with autumn mowing reached their maximum in late August. Consequently, for perennial grasses, the early and late NDVI maxima were analyzed separately. For the first NDVI maximum, the average values ranged from 0.77 to 0.83 for Meteor, 0.76 to 0.83 for Landsat, and slightly lower values, 0.74 to 0.81, for Sentinel. The greatest variability in the first maximum values was observed for Landsat in 2024, with a variation of up to 16.5%, while Meteor composites showed the lowest variation of only 2.8% in 2022. For Sentinel, the timing of the first maximum remained relatively consistent across years (DOY 174–176, p_ANOVA_ > 0.05). However, for Landsat, the first maximum in 2024 was significantly delayed, occurring at DOY 189, compared to 2022–2023 (*p* < 0.0001). The most recent first maximum for perennial grasses was observed in 2023, on DOY 199, which was significantly later than in 2022 and 2024 (*p* < 0.0001). Significant variations in the second NDVI maximum were observed across datasets and years. For Sentinel, values ranged from 0.66 in 2024 (0.1 less than the first maximum) to 0.85 in 2023 (0.1 higher than the first maximum), with statistically significant differences across these years (p_ANOVA_ < 0.0001). The lowest maximum in 2024 also occurred significantly earlier than in 2022–2023 (*p* < 0.0001). For Landsat, the second maximum was observed in 2022 (0.78), with the earliest second maximum recorded on DOY 189, which was significantly earlier than in 2023 and 2024 (*p* < 0.0001). For Meteor, the lowest second NDVI maximum (0.71) and the earliest occurrence (DOY 222) were observed in 2024 (*p* < 0.0001). The detailed NDVI time series characteristics for perennial grasses observed by different satellite systems during the 2022–2024 period are summarized in Table 5 and Table 6.

Table 7 presents the ${NDVI}_{max}$ values for buckwheat fields during 2022–2024. A shift in sowing dates in 2022 resulted in earlier attainment of peak NDVI values, with elevated ${NDVI}_{max}$ levels ranging from 0.83 to 0.88 across different satellite systems. In that year, The NDVI maximum was observed as early as the second half of July, specifically on DOY 196 for Landsat and Sentinel, and on 210 for Meteor. The lowest value of $\bar{{NDVI}_{max}}$ was recorded in 2023, ranging from 0.76 to 0.8, with the peak occurring during the second half of August, on DOY 227 for Landsat and Meteor, and on DOY 242 for Sentinel. Across all three years, Meteor consistently showed the lowest $\bar{{NDVI}_{max}}$ values for buckwheat crops: 0.76 in 2023–2024 and 0.83 in 2022. The ANOVA results confirmed the observed differences in ${NDVI}_{max}$ across years were statistically significant for Sentinel, Landsat, and Meteor (*p* < 0.001).

**Figure 8:**
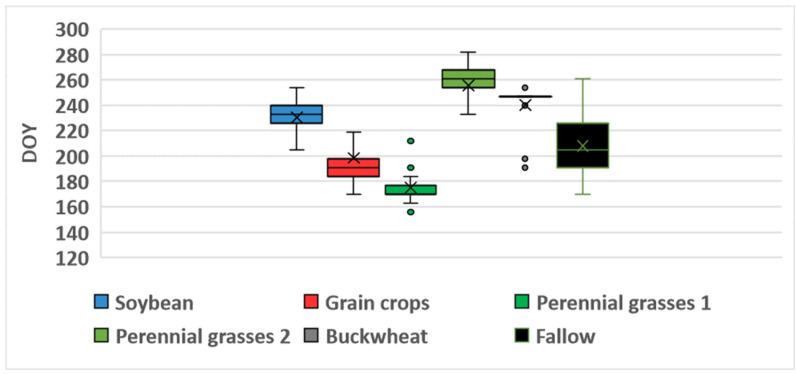
Boxplots for Sentinel-based ${DOY}_{max}$ in 2022–2024.

**Figure 9:**
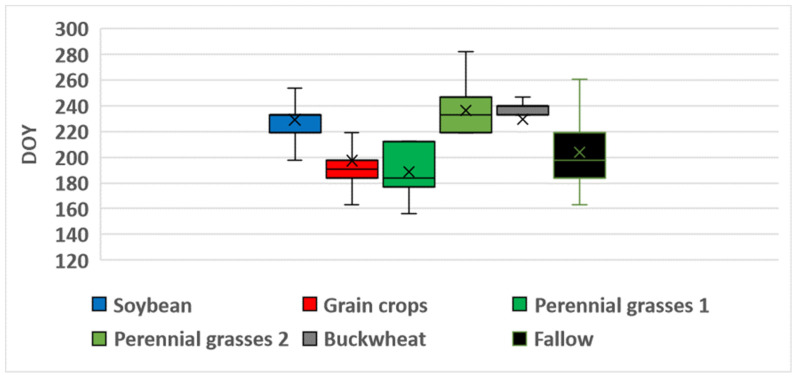
Boxplots for Landsat-based ${DOY}_{max}$ in 2022–2024.

**Figure 10:**
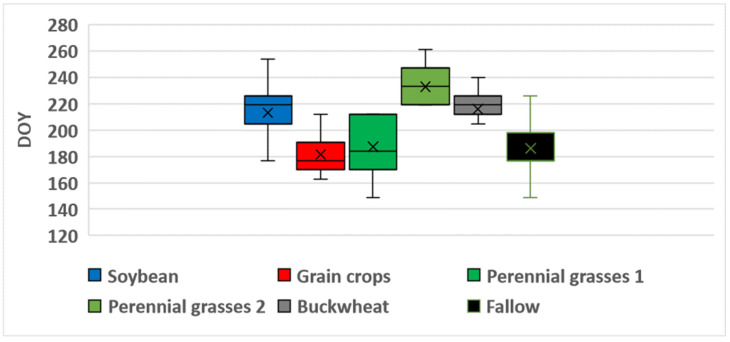
Boxplots for Meteor-based ${DOY}_{max}$ in 2022–2024.

Table 8 summarizes the characteristics of the NDVI time series for unused fields (fallow land) based on satellite data from 2022 to 2024. The highest values of $\bar{{NDVI}_{max}}$ were observed in Landsat data, ranging from 0.87 in 2022 to 0.81 in 2023), with a relatively high variation of 9–12% in 2023 and 2024. In contrast, Sentinel data yielded the lowest $\bar{{NDVI}_{max}}$ values, which ranged from 0.75 in 2023 to 0.77 in 2024. Overall, the highest NDVI values were observed in unused fields in 2022 (0.84–0.87), with the maximum reached during the first half of July (DOY 186–196). Results of the one-way ANOVA confirmed that the value of ${NDVI}_{max}$ in 2022 was significantly higher and occurred earlier than in other years (*p* < 0.0001). In addition, no statistically significant differences in ${NDVI}_{max}$ were found between 2023 and 2024 (*p* > 0.05). Figure 8, Figure 9 and Figure 10 present boxplots of ${DOY}_{max}$ for various crop types and fallow land, derived from Sentinel, Landsat, and Meteor satellite data. These plots clearly demonstrate the differences in ${DOY}_{max}$ for different crops, as well as the similarity in NDVI patterns across different satellites. On average, the earliest ${NDVI}_{max}$ was observed for grain crops, with a $\bar{{DOY}_{max}}$ of 197–198 for both Landsat and Sentinel, and a variation of 23–26 days. In contrast, perennial grasses exhibited significant variability in ${DOY}_{max}$, with standard deviations of 40 days for Landsat and 43 days for Sentinel. The ${NDVI}_{max}$ value could occur either in late June (first maximum) or in early September (second maximum). Both the timing and the number of harvests varied. For fallow land, the peak NDVI value was typically reached in the third decade of July, with a standard deviation of 20–22 days). NDVI heatmaps, showing the dynamics of NDVI values and allowing to estimate the values and time of reaching the maximum, is presented in Figures S1–S3.

Figure 8 shows that, for 33 of 43 buckwheat fields monitored over three years, Sentinel recorded ${NDVI}_{max}$ on DOY 247 (early September), with 2022 as an exception, showing a peak in mid-July. On average, ${NDVI}_{max}$ for Landsat occurred 11 days earlier than for Sentinel and exhibited greater variability. For soybean, the ${NDVI}_{max}$ value was typically reached around DOY 229–230 (mid-August), with a low variation of approximately 10 days, for both Landsat and Sentinel data. In general, Meteor composites exhibited an earlier ${NDVI}_{max}$ across all classes, by an average of 10–20 days, compared to the fitted NDVI curves from Sentinel and Landsat. The only exception was for perennial grasses, where the timing of the Landsat-derived maximum aligned closely with Meteor observations. In addition, the variation in ${DOY}_{max}$ was consistently lower for Meteor than for Landsat and Sentinel, ranging from 11 days for grain crops to 34 days for perennial grasses.

### 3.2. Mapping Results

NDVI time series constructed using three satellite systems were used for classification using the RF algorithm from 2022 to 2024. Table 9, Table 10, Table 11 and Table 12 present the confusion matrices, including F1 scores for each class and OA, for each satellite individually and for the multi-sensor approach. Among the individual satellite datasets, the Landsat-based classification yielded the lowest accuracy (Table 9), with OA declining from 94% in 2022 to 90% in 2023, and further to 84% in 2024. Considering the varying sample sizes across years, the weighted OA was 87%. The F1 score for individual classes was less than 0.80 for buckwheat, perennial grasses, and fallow land, and 0.80 for grain crops. The highest performance was recorded for the most common class, soybean, with an F1 score of 0.94 overall, ranging from 0.91 in 2023 to 0.97 in 2022. Classification accuracy using Meteor data was 2% higher than that obtained using Landsat data, with values of 93% in 2022, 90% in 2023, and 87% in 2024. F1 scores for three classes were also higher: 0.85 for fallow land, 0.86 for grain crops (an improvement of 0.06 over Landsat), and 0.95 for soybean, with annual scores ranging from 0.93 in 2024 to 0.98 in 2022. However, a significant decline in classification accuracy was observed for buckwheat, with an F1 score of only 0.65, with a consistent decrease of over 0.1 each year.

The highest classification accuracy was achieved using the Sentinel NDVI time series, with OA values of 93% in 2023 and 94% in both 2022 and 2024. Meanwhile, F1 scores for all crop classes derived from Sentinel data were higher than those obtained from Landsat and Meteor. Specifically, the F1 score for soybean was 0.96, ranging from 0.95 to 0.97 across three years, 0.93 for grain crops, 0.89 for fallow land, 0.86 for buckwheat, and 0.79 for perennial grasses.

The multi-sensor NDVI-based approach improved classification accuracy by 1% compared to using Sentinel data alone, achieving an OA of 94% across the three years (with 96% in 2022, 95% in 2023, and 94% in 2024). The highest performance was observed for soybeans (F1 = 0.98) and grain crops (F1 = 0.94). The mapping accuracy for buckwheat increased substantially—by 0.06 in 2022 and 2024, and by 0.18 compared to Sentinel alone—resulting in an OA of at least 94% each year. The classification accuracies for perennial grasses and fallow were similar to those obtained using Sentinel data, averaging 0.79 and 0.89, respectively.

Table 13 presents the cross-validation results for crops and fallow land classification from 2022 to 2024, using NDVI time series from three individual satellite systems and a combined approach. In 2022, all four methods demonstrated comparable OA. The highest OA of 97% was achieved by the multi-sensor approach. The mean F1 score (${F1}_{mean}$) was also higher for the combined approach and Sentinel, outperforming Meteor and Landsat. In 2023, the multi-sensor method again achieved the highest performance, with an OA of 96% and an ${F1}_{mean}$ of 0.92. Landsat showed the weakest results, with an OA of 89% and an ${F1}_{mean}$ of 0.75. In 2024, the highest accuracy was obtained using Sentinel and the combined approach, with OA ranging from 92% to 93% and ${F1}_{mean}$ values between 0.87 and 0.88.

**Figure 11:**
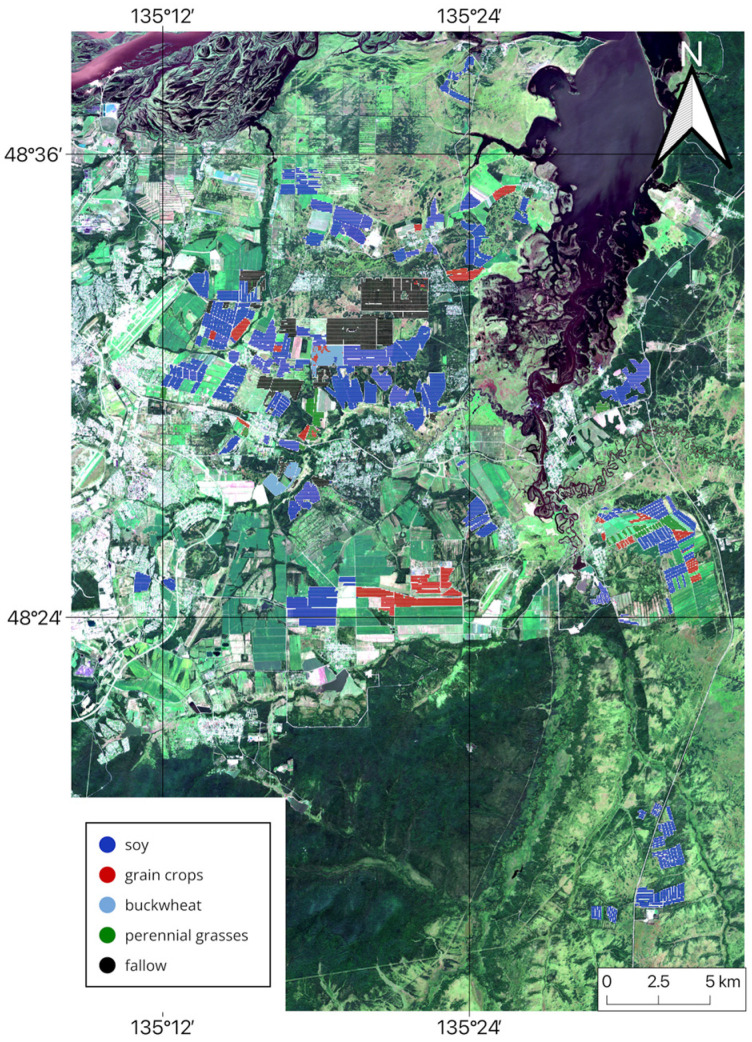
Multi-sensor-based cropland map for Khabarovsk District, 2022.

**Figure 12:**
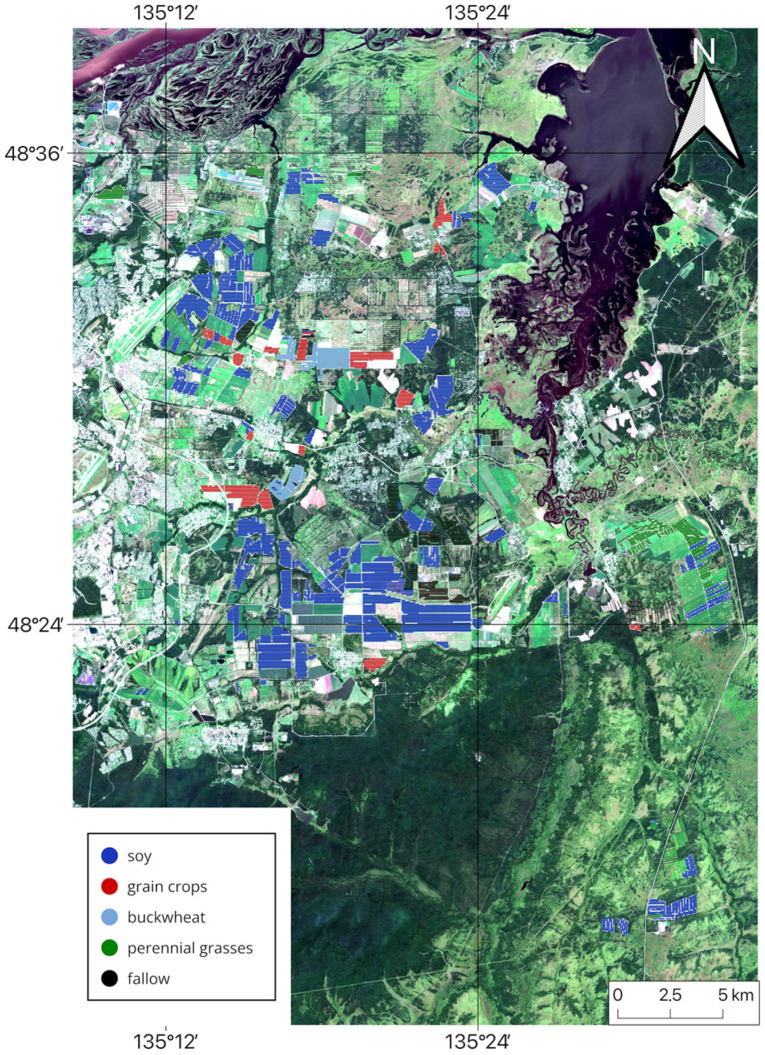
Multi-sensor-based cropland map for Khabarovsk District, 2023.

**Figure 13:**
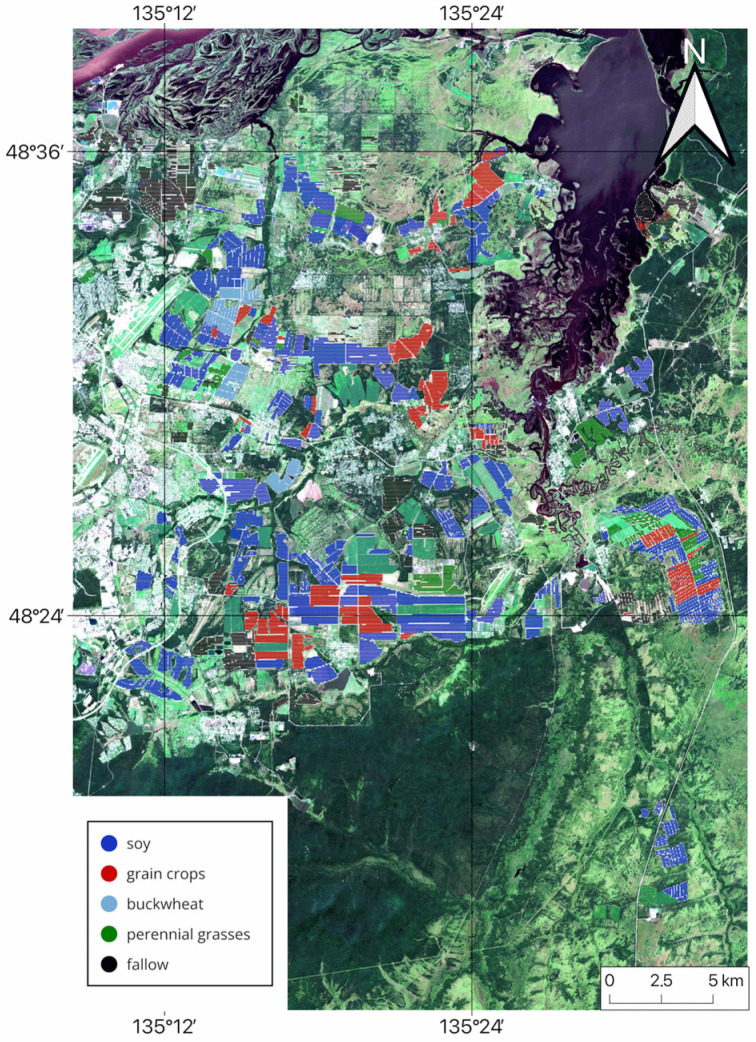
Multi-sensor-based cropland map for Khabarovsk District, 2024.

Thus, the analysis of classification results demonstrates that the multi-sensor approach is highly efficient and enables the creation of accurate cropland maps. Based on the findings of this study, cropland maps for the Khabarovsk district from 2022 to 2024 were generated using NDVI data from three satellites and the RF classification method. These maps indicate the dominant crop type for each field (see Figure 11, Figure 12 and Figure 13).

## 4. Discussion

The southern part of Khabarovsk Krai borders northeastern China (Heilongjiang Province) and shares similar climatic conditions and crop species composition. Several studies conducted by Chinese researchers have mapped crops in these northeastern provinces. Therefore, to evaluate the effectiveness of the approach proposed in this study, it is essential to compare the classification accuracy with prior findings. For instance, the classification of corn, rice, and soybean was conducted in Heilongjiang Province during 2017–2018 using a combination of Sentinel-2 and GaoFen-1 imagery [45]. The datasets were matched to a single grid with a spatial resolution of 16 m. A total of 31 vegetation indices were calculated, and unified time series were constructed by interpolating missing values. The RF and SVM algorithms were applied for classification. Similarly to the findings of our study, classification accuracies were consistently higher when using data from two satellites instead of one. The F1 score for soybean mapping using dual-satellite data was 0.91 in 2017 and 0.86 in 2018, which are significantly lower than the F1 scores obtained using our three-satellite method, which ranged from 0.97 in 2024 to 1.0 in 2022.

In a related study, Zhi et al. performed crop classification in Jilin Province in northeastern China using MODIS and Landsat imagery [46]. Both annual and multiyear datasets were produced by creating weekly composites of various vegetation indices. The F1 score for soybeans in 2019 was relatively low—0.7 using data from 2019 and 0.69 when trained on three-year datasets. These results again demonstrate that the accuracy of soybean mapping in our study exceeds that of previous methods, including those using multi-satellite data. Consequently, the approach proposed in our study may be effectively used in northeastern China, where soybean is a major crop.

In our study, the combined classifier was trained using weekly NDVI composites from three satellites. An alternative strategy is to perform classification separately for each satellite dataset and then combine the results. For example, a recent study in Iran combined machine learning and principal component analysis (PCA), proposing a multi-step Parallel PCA-Cascaded technique for the identification of crops such as wheat, corn, garlic, beet, and alfalfa [47]. Sources of satellite imagery included Sentinel-1, Sentinel-2, and Landsat-8/9. The authors calculated NDVI, EVI, SAVI, NDWI, and Normalized Difference Built-up Index (NDBI) from optical data, along with individual spectral bands. Initial classification was performed on five datasets comprising monthly composites from October 2021 to November 2022: Sentinel-2 bands, Sentinel-2 indices, Landsat bands, Landsat indices, and Sentinel-1 VV (vertical-vertical) and VH (vertical-horizontal) polarizations. The RF classification results (five sets of probabilities for each class) were then used as input for a second machine learning model. This two-step approach achieved a classification accuracy of 92%, which increased to 96% when PCA was applied for component selection. This differs slightly from the accuracy achieved in our study, where cross-validation accuracy for 2022–2023 also ranged from 96 to 97%. However, a key limitation of this approach is the need to calculate and process a large volume of satellite data, including multiple bands and indices, followed by PCA. In contrast, our approach relies only on NDVI values, making it more reproducible and easier to implement.

However, there are two main limitations of study, primarily related to the specific features of the Meteor-M data. First, the data on the Meteor-M channels were only in the form of ready daily composite products. The original imagery for 2022 and 2023 are not available, and the second-level processing data (with a product quality mask like the SCL for Sentinel) are missing for 2024. This made it impossible to apply our fitting algorithm based on Fourier series to the Meteor-M data. At the same time, the Space Research Institute used a local weighted regression method based on the LOWESS approach [48] when creating composites. Differences in the data recovery method led to differences in the seasonal NDVI curves. The second serious limitation of Meteor-M data is the spatial resolution of 60 m, which greatly affects the classification accuracy for small fields. The accuracy for fields of 1–2 hectares (using the example of 2024) was 75%, which is substantially lower than the cross-validation accuracy for all fields in 2024 (87%). These limitations confirm the need to use Meteor-M data in combination with data from other satellite systems.

## 5. Conclusions

Despite the recent success of remote sensing and machine learning methods in cropland mapping and classification, challenges remain in achieving sufficient and stable accuracy across multiple years. In this study, a fitting approach was employed to fit the NDVI time series derived from Landsat and Sentinel imagery. These time series were then used to construct classification models. Additionally, NDVI composites from Meteor data were incorporated. This study evaluated the seasonal stability of NDVI for the primary vegetation classes in the agricultural fields of Khabarovsk Krai over the period 2022–2024. The results revealed that NDVI patterns were consistent across all three satellite systems, indicating their reliability for the classification of arable land. The overall classification accuracy for each year ranged from 84% to 94% for Landsat, 87% to 93% for Meteor, and 93% to 94%. for Sentinel. For the dominant land cover types, i.e., soybean and fallow land, the F1 scores were 0.94 and 0.79 for Landsat, 0.95 and 0.85 for Meteor, and 0.96 and 0.89 for Sentinel, respectively. It should be noted that the classification accuracy using data from the Russian Meteor-M satellite with a resolution of 60 m was higher than for Landsat data (with a resolution of 30 m). While each satellite provided high accuracy individually, combining data from all three satellites further improved the accuracy and stability of the result. The OA of the integrated model ranged from 94% in 2024 to 96% in 2022, with F1 scores of 0.98 for soybean and 0.89 for fallow land. The overall cross-validation accuracy based on Landsat data ranged from 85% to 95%, for Meteor-M—from 87% to 95% based on Sentinel-2 data—from 92% to 96%. The approach proposed in this work, based on combining data from three satellites, allowed to increase the accuracy to 97% in 2022 and to 96% in 2023. These findings demonstrate the efficacy of employing multi-sensor data, particularly in regions affected by frequent cloud cover, which can limit data availability from individual satellites. The consistent and highly accurate results of this approach offer enable the creation of high quality, reliable crop maps, clarification of boundaries of agricultural fields, assessment of utilization of agricultural land, compliance with crop rotations. Solving these problems is valuable for agribusinesses and governmental entities involved in developing agricultural practices and land use policies. This approach can be adapted for the construction of crop maps in other regions, especially for regions with similar climatic conditions and varietal composition. In further studies it is planned to evaluate the use of high spatial resolution data to clarify the boundaries of agricultural fields and identify heterogeneities of vegetation within one agricultural field, to use different vegetation indices to solve different tasks of agricultural monitoring.

## Figures and Tables

**Table 1:** Area of selected crop type classes in the study area (2022–2024).

Year	Class				
	Soybean	Grain Crops	Buckwheat	Perennial Grasses	Fallow Land
2022	4537.52	762.72	260.12	294.80	1422.84
2023	4510.20	662.48	316.04	559.16	773.36
2024	11,336.36	3195.64	845.92	1769.92	4804.96
Overall	20,384.08	4620.84	1422.08	2623.88	7001.16

**Table 2:** Number of images by percentage of masked pixels.

Year	2022		2023		2024	
Masked Pixels, %	Sentinel	Landsat	Sentinel	Landsat	Sentinel	Landsat
<5 (cloud-free)	8	2	11	1	11	2
5–20 (low-cloud)	5	1	4	3	3	2
20–50	11	5	6	3	5	3
>50	37	7	40	7	40	11
Overall	61	15	61	14	59	18

**Table 3:** NDVI time series indicators for soybean fields during 2022–2024.

Satellite	Indicators	2022	2023	2024
Sentinel	$\bar{{NDVI}_{max}}\pm ∆\bar{{NDVI}_{max}}$	0.88 ± 0.01	0.89 ± 0.01	0.87 ± 0.01
	${VAR}_{NDVI}$	4.89	5.12	7.20
	*p* value	p_ANOVA_ < 0.01; p_22–23_ > 0.05, p_22–24_ > 0.05, p_23–24_ < 0.01		
	$\bar{{DOY}_{max}}\pm \Delta \bar{{DOY}_{max}}$	220.77 ± 0.75	231.29 ± 0.72	236.38 ± 0.60
	${VAR}_{DOY}$	2.88	2.35	2.67
	*p* value	p_ANOVA_ < 0.001; p_22–23_ < 0.0001, p_22–24_ < 0.0001, p_23–24_ < 0.0001		
Landsat	$\bar{{NDVI}_{max}}\pm ∆\bar{{NDVI}_{max}}$	0.87 ± 0.01	0.91 ± 0.01	0.87 ± 0.01
	${VAR}_{NDVI}$	5.31	6.29	6.79
	*p* value	p_ANOVA_ < 0.001; p_22–23_ < 0.0001, p_22–24_ > 0.05, p_23–24_ < 0.0001		
	$\bar{{DOY}_{max}}\pm \Delta \bar{{DOY}_{max}}$	223.47 ± 0.87	226.06 ± 1.60	233.90 ± 0.80
	${VAR}_{DOY}$	3.06	5.35	3.64
	*p* value	p_ANOVA_ < 0.001; p_22–23_ < 0.001, p_22–24_ < 0.0001, p_23–24_ < 0.0001		
Meteor	$\bar{{NDVI}_{max}}\pm ∆\bar{{NDVI}_{max}}$	0.88 ± 0.00	0.81 ± 0.01	0.80 ± 0.00
	${VAR}_{NDVI}$	1.97	4.96	3.95
	*p* value	p_ANOVA_ < 0.001; p_22–23_ < 0.0001, p_22–24_ < 0.0001, p_23–24_ <0.0001		
	$\bar{{DOY}_{max}}\pm \Delta \bar{{DOY}_{max}}$	216.57 ± 1.13	229.07 ± 1.22	202.66 ± 1.46
	${VAR}_{DOY}$	4.40	3.99	7.64
	*p* value	p_ANOVA_ < 0.001; p_22–23_ < 0.0001, p_22–24_ < 0.0001, p_23–24_ < 0.0001		

**Table 4:** NDVI time series indicators for grain crops fields during 2022–2024.

Satellite	Indicators	2022	2023	2024
Sentinel	$\bar{{NDVI}_{max}}\pm ∆\bar{{NDVI}_{max}}$	0.77 ± 0.01	0.75 ± 0.03	0.76 ± 0.01
	${VAR}_{NDVI}$	5.09	9.40	8.59
	*p* value	p_ANOVA_ > 0.05		
	$\bar{{DOY}_{max}}\pm \Delta \bar{{DOY}_{max}}$	191.95 ± 8.63	191.00 ± 4.02	203.60 ± 4.48
	${VAR}_{DOY}$	14.79	5.54	11.09
	*p* value	p_ANOVA_ < 0.01; p_22–23_ > 0.05, p_22–24_ < 0.05, p_23–24_ < 0.05		
Landsat	$\bar{{NDVI}_{max}}\pm ∆\bar{{NDVI}_{max}}$	0.80 ± 0.02	0.83 ± 0.03	0.82 ± 0.02
	${VAR}_{NDVI}$	6.98	8.77	9.91
	*p* value	p_ANOVA_ > 0.05		
	$\bar{{DOY}_{max}}\pm \Delta \bar{{DOY}_{max}}$	190.68 ± 9.46	188.34 ± 4.29	203.88 ± 5.12
	${VAR}_{DOY}$	15.08	5.99	12.67
	*p* value	p_ANOVA_ < 0.001; p_22–23_ > 0.05, p_22–24_ < 0.01, p_23–24_ < 0.05		
Meteor	$\bar{{NDVI}_{max}}\pm ∆\bar{{NDVI}_{max}}$	0.83 ± 0.01	0.78 ± 0.02	0.79 ± 0.01
	${VAR}_{NDVI}$	3.11	6.50	5.23
	*p* value	p_ANOVA_ < 0.0001; p_22–23_ < 0.0001, p_22–24_ < 0.0001, p_23–24_ > 0.05		
	$\bar{{DOY}_{max}}\pm \Delta \bar{{DOY}_{max}}$	194.18 ± 1.60	195.10 ± 2.72	172.73 ± 1.06
	${VAR}_{DOY}$	4.00	3.65	3.10
	*p* value	p_ANOVA_ < 0.001; p_22–23_ > 0.05, p_22–24_ < 0.0001, p_23–24_ < 0.0001		

**Table 5:** NDVI time series indicators for fields with perennial grasses during 2022–2024 (first maximum).

Satellite	Indicators	2022	2023	2024
Sentinel	$\bar{{NDVI}_{max}}\pm ∆\bar{{NDVI}_{max}}$	0.81 ± 0.02	0.74 ± 0.02	0.76 ± 0.02
	${VAR}_{NDVI}$	7.0	9.0	11.5
	*p* value	p_ANOVA_ < 0.001; p_22–23_ < 0.001, p_22–24_ < 0.01, p_23–24_ > 0.05		
	$\bar{{DOY}_{max}}\pm \Delta \bar{{DOY}_{max}}$	174.0 ± 3.9	174.6 ± 4.2	176.1 ± 3.2
	${VAR}_{DOY}$	5.9	10.1	7.9
	*p* value	p_anova_ > 0.05		
Landsat	$\bar{{NDVI}_{max}}\pm ∆\bar{{NDVI}_{max}}$	0.83 ± 0.02	0.78 ± 0.01	0.76 ± 0.03
	${VAR}_{NDVI}$	6.3	7.3	16.5
	*p* value	p_ANOVA_ < 0.01; p_22–23_ > 0.05, p_22–24_ < 0.0001, p_23–24_ > 0.05		
	$\bar{{DOY}_{max}}\pm \Delta \bar{{DOY}_{max}}$	173.6 ± 2.8	168.0 ± 1.4	188.7 ± 4.8
	${VAR}_{DOY}$	3.9	3.5	11.4
	*p* value	p_ANOVA_ < 0.0001; p_22–23_ > 0.05, p_22–24_ < 0.0001, p_23–24_ < 0.001		
Meteor	$\bar{{NDVI}_{max}}\pm ∆\bar{{NDVI}_{max}}$	0.83 ± 0.01	0.78 ± 0.01	0.77 ± 0.01
	${VAR}_{NDVI}$	2.8	3.6	5.8
	*p* value	p_ANOVA_ < 0.0001; p_22–23_ < 0.0001, p_22–24_ < 0.01, p_23–24_ > 0.05		
	$\bar{{DOY}_{max}}\pm \Delta \bar{{DOY}_{max}}$	185.2 ± 2.8	199.1 ± 3.6	178.4 ± 3.7
	${VAR}_{DOY}$	4.0	7.6	9.4
	*p* value	p_ANOVA_ < 0.001; p_22–23_ < 0.0001, p_22–24_ > 0.05, p_23–24_ < 0.0001		

**Table 6:** NDVI time series indicators for fields with perennial grasses during 2022–2024 (second maximum).

Satellite	Indicators	2022	2023	2024
Sentinel	$\bar{{NDVI}_{max}}\pm ∆\bar{{NDVI}_{max}}$	0.78 ± 0.04	0.85 ± 0.02	0.66 ± 0.03
	${VAR}_{NDVI}$	12.0	11.2	19.6
	*p* value	p_ANOVA_ < 0.0001; p_22–23_ < 0.01, p_22–24_ < 0.0001, p_23–24_ < 0.0001		
	$\bar{{DOY}_{max}}\pm \Delta \bar{{DOY}_{max}}$	264.7 ± 3.3	261.1 ± 1.7	246.8 ± 4.6
	${VAR}_{DOY}$	3.2	2.6	8.0
	*p* value	p_ANOVA_ < 0.0001; p_22–23_ > 0.05, p_22–24_ < 0.0001, p_23–24_ < 0.0001		
Landsat	$\bar{{NDVI}_{max}}\pm ∆\bar{{NDVI}_{max}}$	0.78 ± 0.03	0.71 ± 0.03	0.73 ± 0.02
	${VAR}_{NDVI}$	9.4	20.3	12.9
	*p* value	p_ANOVA_ < 0.05; p_22–23_ < 0.05, p_22–24_ > 0.05, p_23–24_ > 0.05		
	$\bar{{DOY}_{max}}\pm \Delta \bar{{DOY}_{max}}$	189.37 ± 13.93	204.80 ± 11.25	206.20 ± 7.45
	${VAR}_{DOY}$	6.9	4.8	7.1
	*p* value	p_ANOVA_ < 0.001; p_22–23_ < 0.0001, p_22–24_ < 0.0001, p_23–24_ < 0.0001		
Meteor	$\bar{{NDVI}_{max}}\pm ∆\bar{{NDVI}_{max}}$	0.79 ± 0.02	0.80 ± 0.01	0.71 ± 0.02
	${VAR}_{NDVI}$	6.3	6.1	9.7
	*p* value	p_ANOVA_ < 0.0001; p_22–23_ > 0.05, p_22–24_ < 0.0001, p_23–24_ < 0.0001		
	$\bar{{DOY}_{max}}\pm \Delta \bar{{DOY}_{max}}$	237.9 ± 5.5	244.0 ± 2.4	221.6 ± 1.3
	${VAR}_{DOY}$	6.1	4.1	2.7
	*p* value	*p*_ANOVA_ < 0.001; *p*_22–23_ < 0.01, *p*_22–24_ < 0.0001, *p*_23–24_ < 0.0001		

**Table 7:** NDVI time series indicators for buckwheat fields during 2022–2024.

Satellite	Indicators	2022	2023	2024
Sentinel	$\bar{{NDVI}_{max}}\pm ∆\bar{{NDVI}_{max}}$	0.88 ± 0.04	0.80 ± 0.03	0.83 ± 0.01
	${VAR}_{NDVI}$	3.87	6.03	4.99
	*p* value	p_ANOVA_ < 0.001, p_22–23_ < 0.001, p_22–24_ < 0.05, p_23–24_ > 0.05		
	$\bar{{DOY}_{max}}\pm \Delta \bar{{DOY}_{max}}$	195.67 ± 3.79	242.80 ± 2.85	247.78 ± 0.94
	${VAR}_{DOY}$	1.85	2.12	1.13
	*p* value	p_ANOVA_ < 0.001; p_22–23_ < 0.001, p_22–24_ < 0.001, p_23–24_ < 0.001		
Landsat	$\bar{{NDVI}_{max}}\pm ∆\bar{{NDVI}_{max}}$	0.87 ± 0.07	0.77 ± 0.07	0.87 ± 0.02
	${VAR}_{NDVI}$	4.90	17.47	5.20
	*p* value	p_ANOVA_ < 0.001; p_22–23_ > 0.05, p_22–24_ > 0.05, p_23–24_ < 0.001		
	$\bar{{DOY}_{max}}\pm \Delta \bar{{DOY}_{max}}$	196.25 ± 5.57	227.40 ± 28.87	234.17 ± 7.08
	${VAR}_{DOY}$	1.78	22.92	8.94
	*p* value	p_ANOVA_ > 0.05		
Meteor	$\bar{{NDVI}_{max}}\pm ∆\bar{{NDVI}_{max}}$	0.83 ± 0.02	0.76 ± 0.02	0.76 ± 0.01
	${VAR}_{NDVI}$	2.13	5.04	3.16
	*p* value	p_ANOVA_ < 0.0001; p_22–23_ < 0.001, p_22–24_ < 0.001, p_23–24_ > 0.05		
	$\bar{{DOY}_{max}}\pm \Delta \bar{{DOY}_{max}}$	209.67 ± 3.79	227.87 ± 4.00	212.00 ± 6.28
	${VAR}_{DOY}$	1.72	3.17	8.75
	*p* value	p_ANOVA_ < 0.01; p_22–23_ < 0.05, p_22–24_ > 0.05, p_23–24_ < 0.01		

**Table 8:** NDVI time series indicators for fallow fields during 2022–2024.

Satellite	Indicators	2022	2023	2024
Sentinel	$\bar{{NDVI}_{max}}\pm ∆\bar{{NDVI}_{max}}$	0.84 ± 0.01	0.75 ± 0.01	0.77 ± 0.01
	${VAR}_{NDVI}$	5.78	6.96	7.02
	*p* value	p_ANOVA_ < 0.001; p_22–23_ < 0.0001, p_22–24_ < 0.0001, p_23–24_ > 0.05		
	$\bar{{DOY}_{max}}\pm \Delta \bar{{DOY}_{max}}$	190.88 ± 2.75	215.53 ± 3.77	209.34 ± 2.54
	${VAR}_{DOY}$	5.62	8.77	10.06
	*p* value	p_ANOVA_ < 0.0001; p_22–23_ < 0.0001, p_22–24_ < 0.0001, p_23–24_ < 0.05		
Landsat	$\bar{{NDVI}_{max}}\pm ∆\bar{{NDVI}_{max}}$	0.87 ± 0.02	0.81 ± 0.01	0.83 ± 0.01
	${VAR}_{NDVI}$	6.12	7.94	7.26
	*p* value	p_ANOVA_ < 0.0001; p_22–23_ < 0.0001, p_22–24_ < 0.0001, p_23–24_ > 0.05		
	$\bar{{DOY}_{max}}\pm \Delta \bar{{DOY}_{max}}$	186.98 ± 2.45	210.79 ± 3.77	204.50 ± 2.56
	${VAR}_{DOY}$	4.13	9.21	11.62
	*p* value	p_ANOVA_ < 0.0001; p_22–23_ < 0.0001, p_22–24_ < 0.0001, p_23–24_ < 0.05		
Meteor	$\bar{{NDVI}_{max}}\pm ∆\bar{{NDVI}_{max}}$	0.85 ± 0.00	0.79 ± 0.01	0.80 ± 0.00
	${VAR}_{NDVI}$	1.94	3.82	3.77
	*p* value	p_ANOVA_ < 0.001; p_22–23_ < 0.0001, p_22–24_ < 0.0001, p_23–24_ > 0.05		
	$\bar{{DOY}_{max}}\pm \Delta \bar{{DOY}_{max}}$	196.28 ± 2.91	210.25 ± 2.38	176.64 ± 1.00
	${VAR}_{DOY}$	5.51	5.62	5.29
	*p* value	p_ANOVA_ < 0.001; p_22–23_ < 0.0001, p_22–24_ < 0.0001, p_23–24_ < 0.0001		

**Table 9:** Confusion matrix and accuracy metrics for Landsat data (2022–2024).

	Soybean	Grain Crops	Buckwheat	Perennial Grasses	Fallow Land	F1 Score
Soybean	146,110	5854	304	517	4086	0.94
Grain crops	794	28,652	111	346	618	0.80
Buckwheat	1811	1406	8261	195	470	0.79
Perennial grasses	878	623	135	11,482	3074	0.71
Fallow land	5538	4906	73	3589	42,988	0.79
OA, %	87					

**Table 10:** Confusion matrix and accuracy metrics for Meteor data (2022–2024).

	Soybean	Grain Crops	Buckwheat	Perennial Grasses	Fallow Land	F1 Score
Soybean	33,785	210	68	43	477	0.95
Grain crops	685	7611	13	205	501	0.86
Buckwheat	520	398	1392	38	171	0.65
Perennial grasses	260	331	105	2956	967	0.71
Fallow land	1008	221	157	438	11,238	0.85
OA, %	89					

**Table 11:** Confusion matrix and accuracy metrics for Sentinel data (2022–2024).

	Soybean	Grain Crops	Buckwheat	Perennial Grasses	Fallow Land	F1 Score
Soybean	242,900	2437	1286	1143	6561	0.96
Grain crops	553	55,675	98	193	2761	0.93
Buckwheat	1982	140	16,548	210	853	0.86
Perennial grasses	2020	717	396	18,211	2082	0.79
Fallow land	3154	1510	266	2665	77,502	0.89
OA, %	93					

**Table 12:** Confusion matrix and accuracy metrics for multi-sensor classification (2022–2024).

	Soybean	Grain Crops	Buckwheat	Perennial Grasses	Fallow Land	F1 Score
Soybean	32,137	202	11	43	161	0.98
Grain crops	100	6995	2	33	296	0.94
Buckwheat	165	17	2333	22	22	0.95
Perennial grasses	64	157	6	2450	269	0.79
Fallow land	646	163	6	708	9222	0.89
OA, %	94					

**Table 13:** Cross-validation results.

Satellite Systems	2022		2023		2024	
	OA, %	F1_mean_	OA, %	F1_mean_	OA, %	F1_mean_
Sentinel	96	0.92	92	0.84	93	0.88
Landsat	95	0.87	89	0.75	85	0.78
Meteor	95	0.88	90	0.81	87	0.80
Landsat + Sentinel + Meteor	97	0.93	96	0.92	92	0.87

## Data Availability

Datasets are available on request due privacy.

## Supplementary Materials

The following supporting information can be downloaded at https://www.mdpi.com/article/10.3390/s25185746/s1, Figure S1: NDVI heatmaps in 2022, Figure S2: NDVI heatmaps in 2023, Figure S3: NDVI heatmaps in 2024.

## References

[1] Hassoun A., Mhlanga D., Rejeb A., Bhat Z., Buheji M., Bigliardi B. (2025). The role of industry 4.0 in global food security: A promising pathway to ending hunger. Smart Agric. Technol..

[2] Bigliardi B., Filippelli S., Pini B., Falch E., Gunduz C.P.B., Hassoun A., Hassoun A. (2024). Chapter 2–Industry 4.0 and food sustainability: Role of automation, digitalization, and green technologies. Food Industry 4.0, Developments in Food Quality and Safety.

[3] Javaid M., Haleem A., Singh R.P., Suman R. (2022). Enhancing smart farming through the applications of Agriculture 4.0 technologies. Int. J. Intell..

[4] Konfo T.R.C., Djouhou F.M.C., Hounhouigan M.H., Dahouenon-Ahoussi E., Avlessi F., Sohounhloue C.K.D. (2023). Recent advances in the use of digital technologies in agri-food processing: A short review. Appl. Food Res..

[5] Berger K., Verrelst J., F’eret J.-B., Wang Z., Wocher M., Strathmann M., Danner M., Mauser W., Hank T. (2020). Crop nitrogen monitoring: Recent progress and principal developments in the context of imaging spectroscopy missions. Remote Sens. Environ..

[6] Karmakar P., Teng S.W., Murshed M., Pang S., Li Y., Lin H. (2024). Crop monitoring by multimodal remote sensing: A review. Remote Sens. Appl. Soc. Environ..

[7] Weiss M., Jacob F., Duveiller G. (2020). Remote sensing for agricultural applications: A meta-review. Remote Sens. Environ..

[8] Hao P., Tang H., Chen Z., Meng Q., Kang Y. (2020). Early-season crop type mapping using 30-m reference time series. J. Integr. Agric..

[9] Wulder M.A., Roy D.P., Radeloff V.C., Loveland T.R., Anderson M.C., Johnson D.M., Healey S., Zhu Z., Scambos T.A., Pahlevan N. (2022). Fifty years of Landsat science and impacts. Remote Sens. Environ..

[10] Chen J., Zhang Z. (2023). An improved fusion of Landsat-7/8, Sentinel-2, and Sentinel-1 data for monitoring alfalfa: Implications for crop remote sensing. Int. J. Appl. Earth Obs. Geoinf..

[11] Soriano-González J., Angelats E., Martínez-Eixarch M., Alcaraz C. (2022). Monitoring rice crop and yield estimation with Sentinel-2 data. Field Crops Res..

[12] Plotnikov D., Kolbudaev P., Matveev A., Proshin A., Polyanskiy I. (2023). Accuracy Assessment of Atmospheric Correction of KMSS-2 Meteor-M #2.2 Data over Northern Eurasia. Remote. Sens..

[13] Polyanskiy I.V., Zhukov B.S., Kondratieva T.V., Prokhorova S.A., Smetanin P.S. (2019). Medium-resolution multispectral satellite imaging system for hygrometeorological spacecraft. Sovrem. Probl. Distantsionnogo Zondirovaniya Zemli Kosm..

[14] Zhukov B.S., Kondratieva T.V., Polyanskiy I.V. (2021). Interannual sensitivity trend of the cameras of the multispectral satellite imaging system KMSS-M on Meteor-M No. 2 spacecraft based on the in-flight calibration in 2015–2020. Sovrem. Probl. Distantsionnogo Zondirovaniya Zemli Kosm..

[15] Kashnitskii A.V., Loupian E.A., Plotnikov D.E., Tolpin V.A. (2023). Analysis of the possibility of using different spatial resolution data for objects monitoring. Sovrem. Probl. Distantsionnogo Zondirovaniya Zemli Kosm..

[16] Kolbudaev P.A., Plotnikov D.E., Loupian E.A., Proshin A.A., Matveev A.M. (2021). The methods and automatic technology aimed at imagery georeferencing, cloud screening, atmospheric and radiometric correction of KMSS-M satellite data. E3S Web Conf..

[17] Vidican R., Mălinaș A., Ranta O., Moldovan C., Marian O., Ghețe A., Ghișe C.R., Popovici F., Cătunescu G.M. (2023). Using Remote Sensing Vegetation Indices for the Discrimination and Monitoring of Agricultural Crops: A Critical Review. Agronomy.

[18] Luo K., Lu L., Xie Y., Chen F., Yin F., Li Q. (2023). Crop type mapping in the central part of the North China Plain using Sentinel-2 time series and machine learning. Comput. Electron. Agric..

[19] Ouzemou J.E., El Harti A., Lhissou R., El Moujahid A., Bouch N., El Ouazzani R., Bachaoui E.M., El Ghmari A. (2018). Crop type mapping from pansharpened Landsat 8 NDVI data: A case of a highly fragmented and intensive agricultural system. Remote Sens. Appl. Soc. Environ..

[20] Bellon B., Begue A., Lo Seen D., Lebourgeois V., Evangelista B.A., Simoes M., Demonte Ferraz R.P. (2018). Improved regional-scale Brazilian cropping systems’ mapping based on a semi-automatic object-based clustering approach. Int. J. Appl. Earth Obs. Geoinf..

[21] Toosi A., Javan F.D., Samadzadegan F., Mehravar S., Kurban A., Azadi H. (2022). Citrus orchard mapping in Juybar, Iran: Analysis of NDVI time series and feature fusion of multi-source satellite imageries. Ecol. Inform..

[22] Griffiths P., Nendel C., Hostert P. (2019). Intra-annual reflectance composites from Sentinel-2 and Landsat for national-scale crop and land cover mapping. Remote Sens. Environ..

[23] Pech-May F., Aquino-Santos R., Rios-Toledo G., Posadas-Durán J.-P.F. (2022). Mapping of Land Cover with Optical Images, Supervised Algorithms, and Google Earth Engine. Sensors.

[24] Zhang H., Kang J., Xu X., Zhang L. (2020). Accessing the temporal and spectral features in crop type mapping using multi-temporal Sentinel-2 imagery: A case study of Yi’an County, Heilongjiang province, China. Comput. Electron. Agric..

[25] Chen Y., Cao R., Chen J., Liu L., Matsushita B. (2021). A practical approach to reconstruct high-quality Landsat NDVI time-series data by gap filling and the Savitzky–Golay filter. ISPRS J. Photogramm. Remote Sens..

[26] Julien Y., Sobrino J.A. (2019). Optimizing and comparing gap-filling techniques using simulated NDVI time series from remotely sensed global data. Int. J. Appl. Earth Obs. Geoinf..

[27] Tang L., Zhao Z., Tang P., Yang H. (2020). SURE-based optimum-length S-G filter to reconstruct NDVI time series iteratively with outliers’ removal. Int. J. Wavelets Multiresolut. Inf. Process..

[28] Chu D., Shen H., Guan X., Li X. (2022). An L1-regularized variational approach for NDVI time-series reconstruction considering inter-annual seasonal similarity. Int. J. Appl. Earth Obs. Geoinf..

[29] Zhang L., Shen M., Liu L., Chen X., Cao R., Dong Q., Chen Y., Chen J. (2025). Refining landsat-based annual NDVImax estimation using shape model fitting and phenological metrics. Ecol. Inform..

[30] Zhou J., Jia L., Menenti M. (2015). Reconstruction of global MODIS NDVI time series: Performance of Harmonic Analysis of Time Series (HANTS). Remote Sens. Environ..

[31] Blickensdörfer L., Schwieder M., Pflugmacher D., Nendel C., Erasmi S., Hostert P. (2022). Mapping of crop types and crop sequences with combined time series of Sentinel-1, Sentinel-2 and Landsat 8 data for Germany. Remote Sens. Environ..

[32] Berra E.F., Fontana D.C., Yin F., Breunig F.M. (2024). Harmonized Landsat and Sentinel-2 Data with Google Earth Engine. Remote Sens..

[33] Tian X., Chen Z., Li Y., Bai Y. (2023). Crop Classification in Mountainous Areas Using Object-Oriented Methods and Multi-Source Data: A Case Study of Xishui County, China. Agronomy.

[34] Claverie M., Ju J., Masek J.G., Dungan J.L., Vermote E.F., Roger J.-C., Skakun S.V., Justice C. (2018). The Harmonized Landsat and Sentinel-2 surface reflectance data set. Remote Sens. Environ..

[35] Shen Y., Zhang X., Tran K.H., Ye Y., Gao S., Liu Y., An S. (2025). Near real-time corn and soybean mapping at field-scale by blending crop phenometrics with growth magnitude from multiple temporal and spatial satellite observations. Remote Sens. Environ..

[36] Erdanaev E., Kappas M., Wyss D. (2022). Irrigated Crop Types Mapping in Tashkent Province of Uzbekistan with Remote Sensing-Based Classification Methods. Sensors.

[37] Wang L., Wang J., Liu Z., Zhu J., Qin F. (2022). Evaluation of a deep-learning model for multispectral remote sensing of land use and crop classification. Crop. J..

[38] Loupian E.A., Proshin A.A., Balashov I.V., Burcev M.A., Kashnitskiy A.V., Tolpin V.A., Mazurov A.A., Matveev A.M., Uvarov I.A. (2018). Center for Collective Usage “IKI-Monitoring” (Organization of Distributed Work with Extra Large Archives of Satellite Data for Solving Scientific and Applied Tasks). Inf. Technol. Remote Sens. Earth-RORSE.

[39] Plotnikov D.E., Kolbudaev P.A., Zhukov B.S., Matveev A.M., Bartalev S.A., Egorov V.A., Kashnitskii A.V., Proshin A.A. (2020). The collection of multispectral KMSS-M (Meteor-M No. 2) satellite data aimed at quantitative assessment of the Earth surface. Sovrem. Probl. Distantsionnogo Zondirovaniya Zemli Kosm..

[40] Ilarionova L., Stepanov A., Fomina E., Dubrovin K., Bordakov A. (2024). Program Complex of Automated Processing of Meteor Satellite Composite Images for Obtaining Seasonal NDVI Time Series for Cropland of Khabarovsk Krai.

[41] Gillies S. (2025). Rasterio Documentation.

[42] Rouault E., Warmerdam F., Schwehr K., Kiselev A., Butler H., Łoskot M., Szekeres T., Tourigny E., Landa M., Miara I. (2024). GDAL.

[43] Dubrovin K., Verkhoturov A., Stepanov A., Aseeva T. (2024). Multi-Year Cropland Mapping Based on Remote Sensing Data: A Case Study for the Khabarovsk Territory, Russia. Remote Sens..

[44] Pedregosa F., Varoquaux G., Gramfort A., Michel V., Thirion B., Grisel O., Blondel M., Prettenhofer P., Weiss R., Dubourg V. (2011). Scikit-learn: Machine Learning in Python. J. Mach. Learn. Res..

[45] Yan S., Yao X., Zhu D., Liu D., Zhang L., Yu G., Gao B., Yang J., Yun W. (2021). Large-scale crop mapping from multi-source optical satellite imageries using machine learning with discrete grids. Int. J. Appl. Earth Obs. Geoinf..

[46] Zhi F., Dong Z., Guga S., Bao Y., Han A., Zhang J., Bao Y. (2022). Rapid and Automated Mapping of Crop Type in Jilin Province Using Historical Crop Labels and the Google Earth Engine. Remote Sens..

[47] Abdali E., Valadan Zoej M.J., Taheri Dehkordi A., Ghaderpour E. (2024). A Parallel-Cascaded Ensemble of Machine Learning Models for Crop Type Classifica-tion in Google Earth Engine Using Multi-Temporal Sentinel-1/2 and Landsat-8/9 Remote Sensing Data. Remote Sens..

[48] Cleveland W.S. (1979). Robust locally weighted regression and smoothing scatterplots. J. Am. Stat. Assoc..

